# Supplementary material for: A coupled human-Earth model perspective on long-term trends in the global marine fishery
Source: Nat Commun. 2017 Mar 27;8:14884. doi: 10.1038/ncomms14884 (PMC5556735; doi:10.1038/ncomms14884)
Supplement: Supplementary Information — Supplementary Figures, Supplementary Table, Supplementary Methods and Supplementary References [file ncomms14884-s1.pdf]

Supplementary Methods

Model description

The ecosystem component of the BiOeconomic mArine Trophic Size-spectrum (BOATS) model takes a macroecological approach, founded on a well-developed body of theory and empirical parameterizations that have been shown to explain many aspects of organisms as a function of size and body temperature. The model is described here briefly; the reader is referred to refs. 1 and 2 for a detailed description.

The configuration of BOATS used here simulates all commercial species as three aggregate size spectra (k=1:3), that differ only in their maximum sizes, chosen to correspond to the groups in the Sea Around Us Project (SAUP) database[3]. These groups are not intended to represent the entire marine ecosystem, but rather the sum of all species that have been commercially harvested (and are therefore accounted for in harvest records, which are used to constrain the model). The underlying philosophy of the model is that, although these very diverse species differ widely in their biological strategies, all are competing for food energy ultimately provided by the fixation of organic carbon through photosynthesis (which has been shown to limit fish harvests, ref. 4), while inhabiting the same environment, which therefore makes them subject to the same metabolic constraints. The constraints we apply in the model are the impacts of water temperature on growth, mortality, and phytoplankton size, and the net primary production. Although this biologically ‘coarse-grained' approach precludes resolution of species-level dynamics, it is solidly-founded in bioenergetic principles, and is well-suited to the global view of the entire ecosystem on long timescales, given that it is likely to be relatively robust under any

changes in the distribution, abundance or evolution of commercial species. Supplementary Figure 1 provides a schematic overview of the model structure.

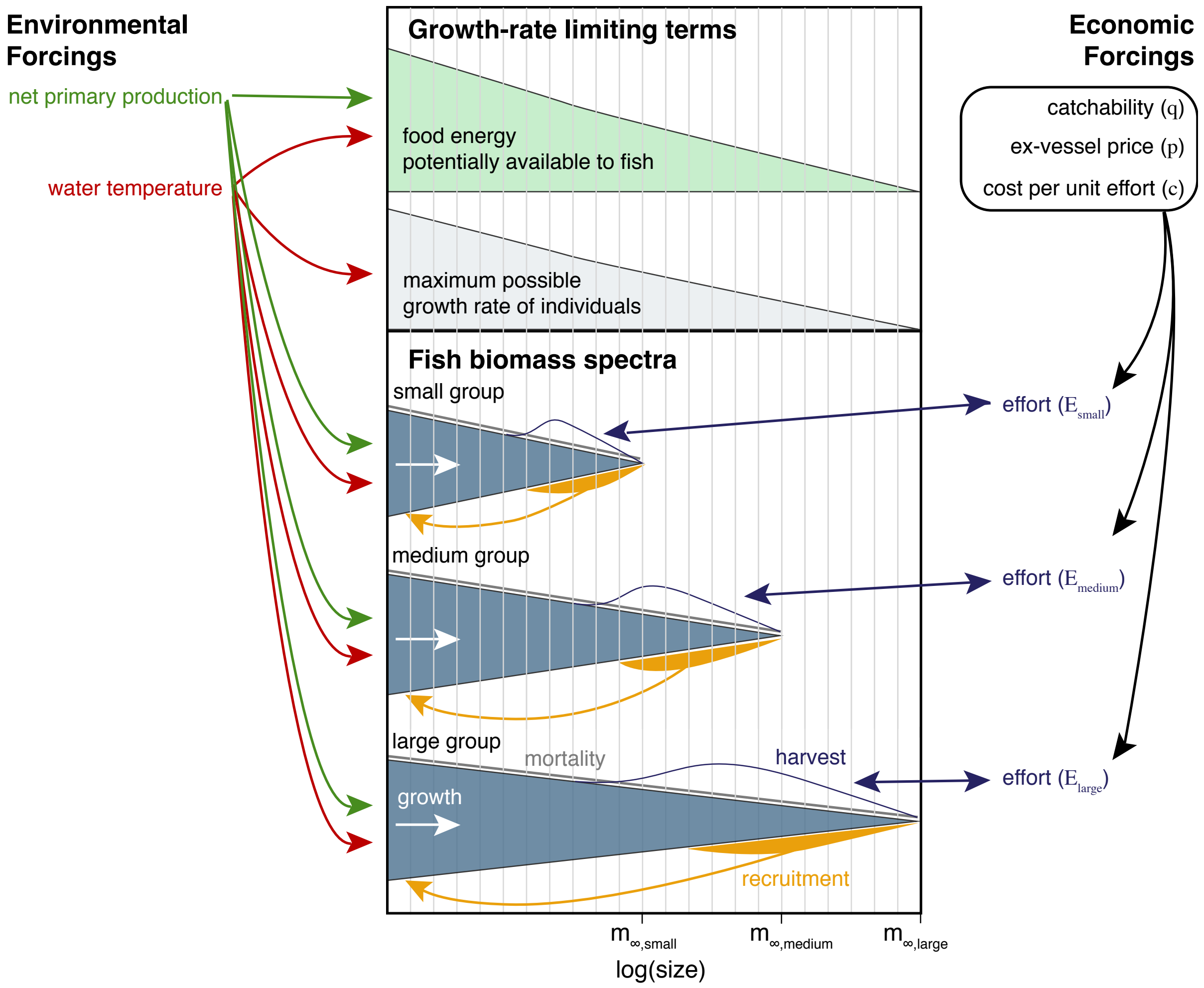

Supplementary Figure 1. Schematic overview of the BOATS model. The red, green, and black arrows indicate dependencies of model components on external forcings. The top panel indicates the energetic limits of growth as a function of fish size, while the bottom panel illustrates the three size spectra of fish groups, their internal dynamics, and link to economics via harvest and the interactive effort.

Each horizontal grid cell represents the vertically-integrated population of potentially-commercial organisms within that grid cell. The growth of ‘fish’, including finfish and invertebrates, within each spectrum is given by the McKendrick-von Foerster equation,

$$\frac{\partial}{\partial t} f_k = -\frac{\partial}{\partial m} \gamma_k f_k + \frac{\gamma_k f_k}{m} - \Lambda_k f_k \qquad (1)$$

where m is a mass element, t is time, f_k is the biomass spectrum per unit area of group k, and Λ_k is the natural mortality rate. The growth rate, γ, for an individual of mass m, is limited by the energy available from photosynthesis in the grid cell, π, which, following ref. 5, is calculated as:

$$\pi = \frac{\Pi}{m}\left(\frac{m}{m}\right)^{\tau-1} \tag{2}$$

where Π_ψ is the local net photosynthesis, τ is a trophic scaling exponent depending on the average trophic efficiency and predator to prey mass ratio[5] that indicates how efficiently photosynthetic energy is transferred through the food web, and m_ψ is the geometric-mean size of phytoplankton cells. Each fish group can potentially access an equal fraction Φ_k of the total primary production energy, representing an exclusive ecological niche. In addition, the growth rate of individuals is limited by a temperature- and size-dependent maximum growth rate,

$$\gamma_{VB} = Am^b - k_a m \tag{3}$$

where A and k_a are temperature-dependent, and b is a constant. This limit represents the biological maximum growth rate when food is not limiting. Thus, the total energy available for growth is

$$\xi_{I,k} = \min\left[\frac{\phi_k \pi m}{f_k}, \gamma_{VB}\right] \tag{4}$$

This energy is then partitioned between somatic growth and reproduction as a function of mass relative to asymptotic mass, following Andersen and Beyer[6].

The bottom boundary condition of each size spectrum is given by a modified Beverton-Holt[7] recruitment relationship that depends on integrated spawner biomass and reproduction rate within the spectrum, the water temperature, and on the availability of food, consistent with observations[8]. Although predicting the recruitment of an individual species in any given year has

proven a vexing problem[9], our model aims for a much more modest goal, which is to predict the total recruitment of all species, classified only by their asymptotic size. Where the biomass production of commercial species is limited by growth rates or egg production, the excess photosynthetic energy is assumed to supply non-commercial species (such as unharvested zooplankton, jellyfish and benthic invertebrates).

The Open Access (OA) economic model is a spatially- and temporally-resolved implementation of the Gorden-Schaefer model[10,11]. Fish harvest of group k, (H_k) is determined in each grid cell, during each time step, as

$$H_k = q\, E_k\, \sigma_k f_k \tag{5}$$

where E_k is the nominal fishing effort applied to the group, σ_k is a function representing the size-selectivity of fishing gear on group k, and q is a catchability parameter that represents the effectiveness of fishing technology in catching the selectable fish biomass. Because the model resolves life history through the size spectrum, the size-selectivity of fishing gear is an important determinant of the impacts of harvest mortality[12,13]. Although different gear types have different size selectivities, it has been shown that most are sigmoidal, with low selectivity for juveniles, transitioning to nearly complete selectivity across a threshold size[14,15]. We therefore define the size-selectivity function as

$$\sigma_k = \left[1 + \left(\frac{m}{m_{T,k}}\right)^{-c_\sigma/\delta_2}\right]^{-1} \tag{6}$$

where the threshold mass $m_{T,k}$ is defined by a fraction of the maturity size, given by a position scaling term e_{mT}, and the parameters c_σ and δ define the shape of the selectivity vs. size.

The fishing effort on each group, E_k, evolves independently in each grid cell over the simulation according to the local average profit, which is the difference between the local revenue (the fish price multiplied by the harvest) and the local cost (the fishing cost multiplied by the effort):

$$\frac{\mathrm{d}}{\mathrm{dt}} E_k(t) = \frac{\kappa_e [\mathrm{revenue}_k - \mathrm{cost}_k]}{E_k(t)} \tag{7}$$

The fleet adjustment parameter κ_e represents the time required for processes such as gaining access to capital, gear purchase, and organization of labour, and is set so that effort responds on a ten-year timescale to changes in profit. Together, this relatively simple representation captures the dynamics of an open access fishery, leading to zero profit at steady state.

The cost per unit effort is assumed to include investment, repair and maintenance of fishing infrastructure, fuel consumption, and labour cost in bringing the fish to market. As a first approximation, costs are assumed to be spatially uniform, and do not account for distance to port. "Price" is that paid to the fishermen at the point of landing (ex-vessel), and is also spatially uniform, though a temporally-variable price is imposed in some experiments. The possibility of using different prices for different fish sizes was explored, but we failed to identify simple, robust relationships between asymptotic size and price that held across all commercial fish over time. For example, anchovies and shrimp have similar asymptotic sizes, but shrimp tend to be far more valuable per kg. These prices seem to reflect societal preference related to factors such as taste, appearance, convenience or cultural value, rather than fundamental features related to the size, and we therefore consider them difficult to predict.

The model is discretized on a 1-degree grid with a monthly time-step and solved numerically.

Parameter optimization

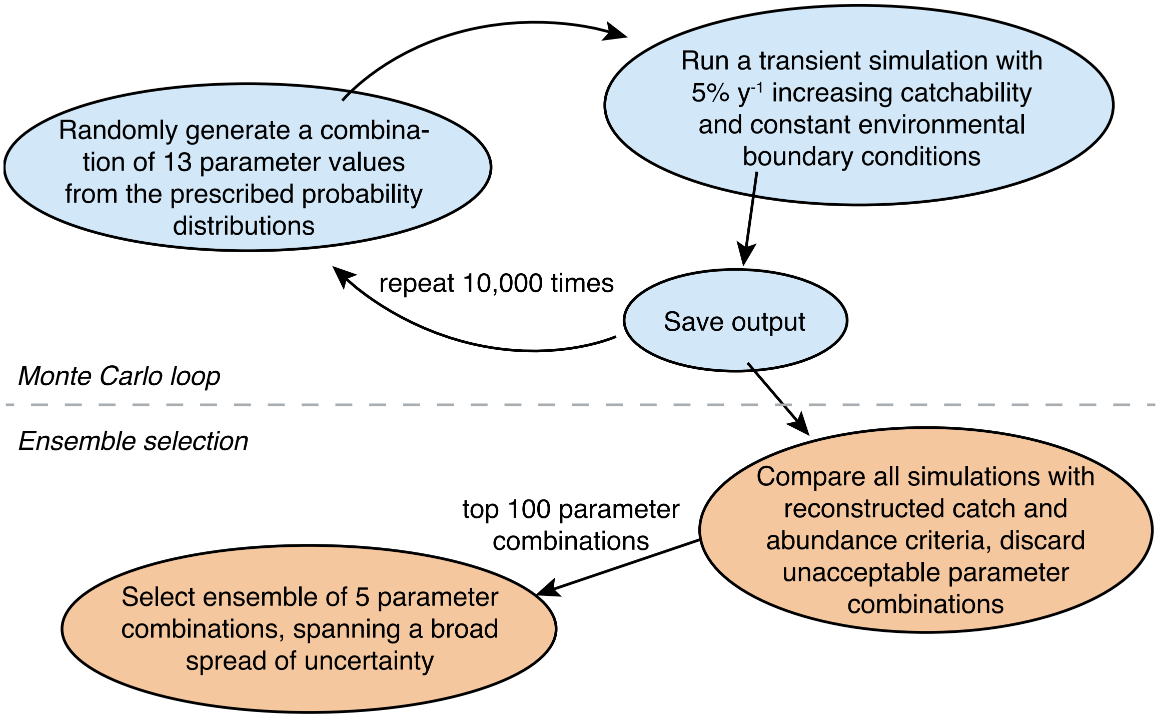

Supplementary Figure 2. Schematic illustration of the parameter selection.

The model parameters were optimized using a Monte Carlo-based approximate Bayesian computation, schematically illustrated in Supplementary Figure 2. First, 10,000 random combinations of parameter values were chosen from within defined probability distributions (Supplementary Table 1), and for each parameter combination the global model was integrated through a 200 year transient with increasing catchability at 5% y^{-1}, starting from a very low value. Parameter combinations were evaluated by comparing each of the 10,000 simulations to catch data provided by the Sea Around Us Project[3] and stock assessment data[16] for 8 LMEs with relatively comprehensive assessments representing >40% of the historically-harvested species (Baltic Sea, Barents Sea, Patagonian Shelf, Benguela Current, North Sea, Okhotsk Sea, Gulf of

Parameter	Name	Sampling distribution	EM1	EM2	EM3	EM4	EM5
$\omega_{a,A}$	Growth activation energy	Normal (0.45, +/- 0.09)	0.31	0.37	0.40	0.43	0.42
$\omega_{a,\lambda}$	Mortality activation energy	Normal (0.45, +/- 0.09)	0.37	0.34	0.64	0.51	0.56
b	Allometric scaling exponent	Uniform (0.61-0.79)	0.67	0.62	0.63	0.69	0.65
A_0	Allometric growth constant	Normal (0.46, +/- 0.5)	0.37	0.40	0.41	0.42	0.40
α	Trophic efficiency	Uniform (0.06-0.20)	0.16	0.16	0.15	0.14	0.18
β	Predator to prey mass ratio	Uniform (675-9330)	7609	1778	8463	7709	8782
k_E	Eppley constant	Normal (0.0631, +/- 0.009)	0.06	0.07	0.05	0.06	0.06
Π^*	Nutrient concentration	Normal (0.37, +/- 0.1)	0.31	0.29	0.20	0.27	0.35
ζ_1	Mortality constant	Normal (0.55, +/- 0.57)	0.27	0.03	0.64	-0.25	0.75
h	Allometric mortality scaling	Normal (0.54, +/- 0.09)	0.6	0.56	0.39	0.51	0.34
s_e	Egg survival fraction	Uniform (0.0001-0.0492)	0.03	0.04	0.01	0.03	0.03
e_{mT}	Selectivity position scaling	Uniform (0.50-1.50)	0.61	1.23	0.54	0.86	0.84
c_σ	Selectivity slope	Uniform (12.0-24.0)	16.7	13.0	20.8	12.4	12.4
		**Correlation (Pearson r^2)**	0.56	0.51	0.54	0.49	0.56

Supplementary Table 1. Model parameter values varied in Monte Carlo optimization procedure. Parameter values were randomly selected from either a normal distribution (mean, 1 s.d.) or a uniform distribution (min-max) as indicated. The values for each of the five ensemble members (EM) are also given. The last row gives the linear correlation between simulated harvests and the SAUP harvests by LME, for each ensemble member.

Mexico, and East Bering Sea). We then sequentially applied the following four criteria in order to discard 99% of the parameter combinations: 1) the total LME peak harvest was required to fall between 70 and 150 Mt y^{-1} (a very broad range, both to account for uncertainty in the reconstructed harvests and in the allocation of primary production to commercial fish); 2) the global LME peak harvest was required to have medium harvest that was at least 30% of the small harvest and large harvest that was between 10 and 80% of the small harvest; 3) the r^2 between simulated and observed peak LME harvest was required to be at least 0.45 (see discussion below on peak harvests); 4) given that the total assessed harvest:biomass ranged from 0.09 to 0.29 y^{-1} among the 8 thoroughly-assessed LMEs, and considering that none of the

assessments include all potentially commercial species and are therefore quite uncertain, the simulated harvest:biomass ratios of these 8 LMEs were simply required to be less than 0.4 y^{-1}. The application of these four metrics resulted in a total of 100 parameter combinations. Of these, we selected five models that span broad ranges in total global harvest and biomass, in order to represent a large swath of parameter uncertainty (Supplementary Table 1).

Peak harvests as a model calibration target

The gradual increase of catchability, q, due to technological progress, produces an inexorable increase of effective effort that causes fish biomass, f, to decrease. Because the harvest (H) is given by $H = q\, E\, \sigma\, f$, (see above), the decrease of f ultimately yields to a decline of harvest, so that a peak in fish harvest occurs at some catchability q_p. The harvest at the q_p peak is therefore a reflection the ability of the ecosystem to produce biomass under a transient increase of fishing pressure[17,18], although with caveats. As such, it can be considered an inherent property of an ecosystem that is relatively insensitive to the rate at which effective effort increases, as long as the timescale at which effort increases is longer than the replacement timescale of fish in the ecosystem.

Therefore, where peak harvests can be estimated from observational data, they provide a constraint on the ecosystem's capacity to produce commercial fish, which is a function of ecosystem parameters, and is relatively independent of socio-economic conditions. Effective management could theoretically prevent yields from ever reaching the ecosystem maximum, but given that management was only instigated prior to ecosystem degradation in a few cases (e.g.

the eastern Bering Sea), it is likely that most LME harvest peaks provide a rough indication of ecosystem productivity. Since our model is defined as simulating all commercial species, the total model harvest is directly comparable to the total observed harvest.

We examined the time series of global Large Marine Ecosystems (LMEs) in the SAUP data to define as many peak harvests as possible. Peaks were defined as the average of the 10 highest years of harvest. Model harvest peaks were simulated by forcing the model with continual transient increases of technology, and the corresponding LMEs of the model simulations were compared with observed harvest peaks at 55 LMEs (see 'Parameter optimization'). Although this method is hampered by errors in the observational estimates of fish harvest, it provides a uniquely comprehensive means to calibrate our model over the full global range of marine environments.

Ex-vessel Price variations

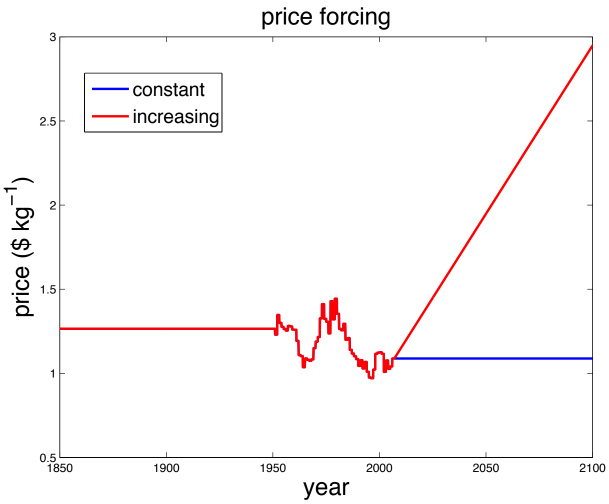

Supplementary Figure 3. Ex-vessel price used in historical simulation (red), future constant price (blue) and future increasing price (red).

The ex-vessel price is taken from ref. 19 for the period 1950-2006. The price is held constant prior to 1950 at the 1950 value. From 2007-2100, it is either held constant at the 2006 value, or increases linearly at a rate of 2 $ kg^{-1} century^{-1} , similar to the rate of price increase between 1960 and 1975 (see Supplementary Figure 3).

Historical global average cost per unit effort

Although there is a global, observationally-based estimate of cost per unit effort[15], it is only available for a recent time interval, and historical variations over time are not resolved. Therefore, in order to test the impact that temporally-variable cost per unit effort might have had on the history of global catches, we conducted a pair of sensitivity tests based on a simple line of reasoning.

The open access model employed here simulates changes in effort, *E*, over time as $dE/dt = (pH-cE)/E$, where *p* is ex-vessel price and *c* is cost per unit effort. At steady state, $pH=cE$ and

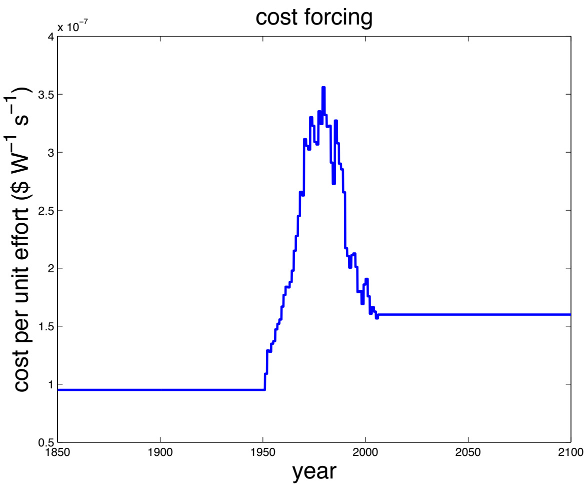

Supplementary Figure 4. Cost per unit effort, reconstructed as described.

therefore $c=pH/E$. Because p, H and E are all available from observational estimates (see Figure 1 of main text), the average cost per unit effort can be easily calculated, assuming that the fishery is always close to steady state (Supplementary Figure 4).

It is important to recognize that the steady state approximation is incorrect, and this calculation is unlikely to provide an accurate history of the global catch per unit effort. Given that, in reality, the adjustment timescale for E is many years, it cannot respond instantaneously to changes in price, cost and biomass, so that our steady state estimate is likely to provide an exaggerated amplitude of changes in cost over time. For this reason, we do not include it in the simulations other than to test the impact of cost alone. Despite these uncertainties, the reconstruction suggests that globally-averaged historical cost changes did not exceed a factor of 3, and does not suggest a monotonic trend.

Hindcast and projection simulation protocol

For all ensemble members, we first conducted a spin-up using the steady pre-industrial environmental forcings but without harvest, to represent a global preindustrial fishing state. We then needed to define a relationship between the transient model simulations and historical years, in order to apply the reconstructed ex-vessel price and variable climate simulations, and to compare directly with data. We did this by using the year of global peak harvest as a reference point, identified in the SAUP database as approximately 1995. We then coordinated each ensemble member with this peak year in two steps. First, starting from the preindustrial fishing state, and using the climatological forcing with constant average ex-vessel prices and cost per

unit effort, we imposed a technology increase of 5% y^{-1}. Then, the model catchability during the peak year was projected backwards using the same 5% y^{-1} assumption to give an idealized, low-catchability starting point in calendar year 1850 while ensuring that the peak global harvest will result in the late 1990s; note that we do not expect this procedure to generate a realistic catchability for 1850, it simply serves as a reasonable starting point, to avoid spurious initialization effects. The year of peak global harvest in the full hindcasts is shifted slightly, due to the effects of historically-variable climate and ex-vessel price. Nonetheless, this simple procedure successfully results in peak harvest years between 1995 and 1996 for the five ensemble members, within the uncertainty of the actual peak in the SAUP harvest data.

Global peak in fish harvest

The simulated global peak in fish harvest, shown in Figure 3, is given by the sum of thousands of individual 'fisheries', each operating at a single grid cell, and each of which goes through the classic periods of development, full exploitation, over-exploitation and collapse[20]. Supplementary Figure 5 shows the aggregates of these individual grid cell harvests at the LME level for the standard hindcast simulation shown in Figure 3 (for the ensemble member with highest r^2). The transient change of harvest in each LME is dominated by technological progress, with small influences from changes in ex-vessel prices and climate variability, while the differences between LMEs reflect differences in environmental characteristics (NPP and water temperature).

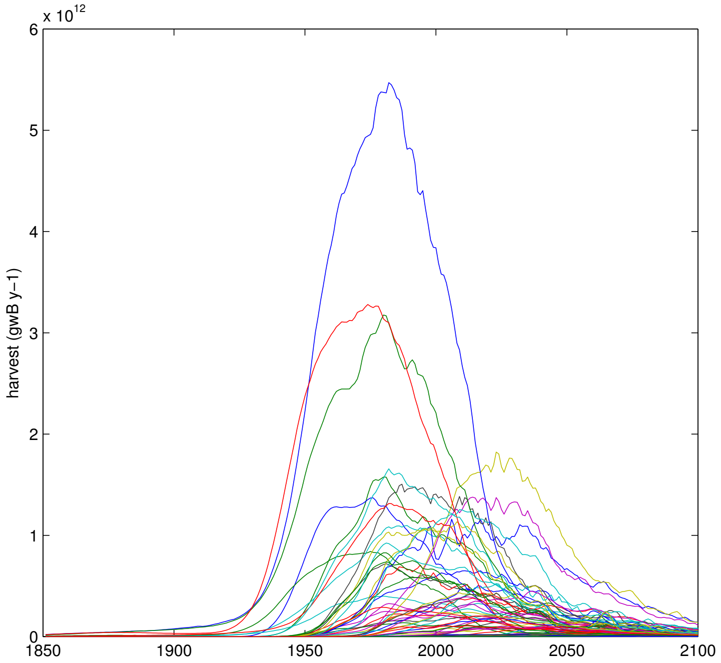

Supplementary Figure 5. Harvest timeseries, aggregated by LME. Each line shows the transient harvest for one LME in the standard hindcast simulation and under the continued 5 % progress with OA (figure 3). Each LME passes through the well-recognized phases of fisheries: a period of development (when harvest is increasing over time), full exploitation (when the harvest reaches a relatively stable peak), over-exploitation (when the harvest starts to decrease) and collapse (when harvest reaches a small fraction of its peak value). The global peak of harvest reflects the sum of these individual ecosystem curves.

Calculation of MSY

Although it has proven to be a problematic term in fisheries science[21,22], Maximum Sustainable Yield is nonetheless a useful concept for considering the maximum harvest that could be perpetually extracted from the marine environment assuming no degradation of the ecosystem. We therefore include an estimate of the model's MSY, as constrained by the optimized ecosystem parameters, in order to approximate the total marine food resource that might be obtained under perfect management intended to maximize food production (rather than economic, cultural or other benefit)[23].

The MSY was estimated for each ensemble member by increasing catchability at a very slow rate (1% y^{-1}) for 1500 years, after a 200-year equilibration with no harvest. This produces a very smooth, gradual peak of harvest in each grid cell of the model, that closely approximates a succession of steady state harvests each in equilibrium with a different level of catchability. The results were then sampled for the peak harvest achieved in each grid cell, and the results summed in order to give the global peak. In order to calculate the MSY under climate change, the same procedure was followed using a climatology of NPP and temperature obtained from the IPSL transient simulation at 10-year intervals. The result, plotted in Figure 5, interpolates between these 10-year snapshots.

Climate change simulations

In order to test the model sensitivity to climate change, we used output from the Institut Pierre Simon Laplace (IPSL) low-resolution (LR) coupled model. This model was chosen for the fact that its response of global Net Primary Production (NPP) to warming is close to the Coupled Model Intercomparison Project phase 5 (CMIP5) multi-model mean[24]. The vertically-integrated primary production and shallow subsurface water temperature were used to force the BOATS model ensemble discussed in the main text. In order to clearly show the impact of climate change, we compare a climatological pre-industrial repeating-year ("preindustrial") with monthly values from the RCP8.5 transient simulation ("RCP8.5").

The difference in harvest between the preindustrial simulation and the RCP8.5 simulation reflect the sensitivity of the BOATS ecosystem model to the changes of NPP and water temperature

predicted by the IPSL climate-biogeochemical model. This occurs through the impact of water temperature on phytoplankton cell size, organismal growth rates and natural mortality, and the overall energy available to the ecosystem from NPP.

Supplementary References

1 Carozza, D. A., Bianchi, D. & Galbraith, E. D. The ecological module of BOATS-1.0: a bioenergetically constrained model of marine upper trophic levels suitable for studies of fisheries and ocean biogeochemistry. *Geosci Model Dev* **9**, 1545-1565 (2016).

2 Carozza, D. A., bianchi, D. & Galbraith, E. Reducing parameter uncertainty in a bioenergetically-constrained ecology-economic model of the global fishery. *Plos One* (in press).

3 Pauly, D. The Sea Around Us Project: Documenting and communicating global fisheries impacts on marine ecosystems. *AMBIO: a Journal of the Human Environment* **36**, 290-295 (2007).

4 Chassot, E. *et al.* Global marine primary production constrains fisheries catches. *Ecology letters* **13**, 495-505 (2010).

5 Brown, J. H., Gillooly, J. F., Allen, A. P., Savage, V. M. & West, G. B. Toward a metabolic theory of ecology. *Ecology* **85**, 1771-1789 (2004).

6 Andersen, K. H. & Beyer, J. E. Size structure, not metabolic scaling rules, determines fisheries reference points. *Fish and Fisheries* **16**, 1-22 (2015).

7 Beverton, R. J. H. & Holt, S. J. On the Dynamics of Exploited Fish Populations. (Springer-Science+Business Media, 1957).

8 Vert-pre, K. A., Amoroso, R. O., Jensen, O. P. & Hilborn, R. Frequency and intensity of productivity regime shifts in marine fish stocks. *Proceedings of the National Academy of Sciences* **110**, 1779-1784 (2013).

9 Houde, E. D. Emerging from Hjort's shadow. *Journal of Northwest Atlantic Fishery Science* **41**, 53-70 (2008).

10 Gordon, H. S. The Economic Theory of a Common-Property Resource: The Fishery. *Journal of Political Economy* **62**, 124-142 (1954).

11 Schaefer, M. B. Some considerations of population dynamics and economics in relation to the management of the commercial marine fisheries. *Journal of the Fisheries Board of Canada* **14**, 669-681 (1957).

12 Jacobsen, N. S., Gislason, H. & Andersen, K. H. The consequences of balanced harvesting of fish communities. *Proceedings of the Royal Society of London B: Biological Sciences* **281**, 20132701 (2014).

13 Andersen, K. H. *et al.* Assumptions behind size-based ecosystem models are realistic. *ICES Journal of Marine Science: Journal du Conseil*, fsv211 (2016).

14 Millar, R. B. & Fryer, R. J. Estimating the size-selection curves of towed gears, traps, nets and hooks. *Reviews in Fish Biology and Fisheries* **9**, 89-116 (1999).

15 Lam, V. W., Sumaila, U. R., Dyck, A., Pauly, D. & Watson, R. Construction and first applications of a global cost of fishing database. *ICES Journal of Marine Science: Journal du Conseil* **68**, 1996-2004 (2011).

16 Ricard, D., Minto, C., Jensen, O. P. & Baum, J. K. Examining the knowledge base and status of commercially exploited marine species with the RAM Legacy Stock Assessment Database. *Fish and Fisheries* **13**, 380-398 (2012).

17 Srinivasan, U. T., Cheung, W. W., Watson, R. & Sumaila, U. R. Food security implications of global marine catch losses due to overfishing. *Journal of Bioeconomics* **12**, 183-200 (2010).

18 Halpern, B. S. *et al.* An index to assess the health and benefits of the global ocean. *Nature* **488**, 615-620 (2012).

19 Sumaila, U. R., Marsden, A. D., Watson, R. & Pauly, D. A global ex-vessel fish price database: construction and applications. *Journal of Bioeconomics* **9**, 39-51 (2007).

20 Kleisner, K., Zeller, D., Froese, R. & Pauly, D. Using global catch data for inferences on the world's marine fisheries. *Fish and Fisheries* **14**, 293-311 (2013).

21 Larkin, P. A. An epitaph for the concept of maximum sustained yield. *Transactions of the American fisheries society* **106**, 1-11 (1977).

22 Mace, P. M. A new role for MSY in single-species and ecosystem approaches to fisheries stock assessment and management. *Fish and fisheries* **2**, 2-32 (2001).

23 Andersen, K. H., Brander, K. & Ravn-Jonsen, L. Trade-offs between objectives for ecosystem management of fisheries. *Ecological Applications* **25**, 1390-1396 (2015).

24 Bopp, L. *et al.* Multiple stressors of ocean ecosystems in the 21st century: projections with CMIP5 models. *Biogeosciences* **10**, 6225-6245 (2013).
